# Improvement of a phylogenetic footprinting method for transcription factor binding sites recognition based on the use of bootstrap trials for the analysis of large bacterial genomic data

**DOI:** 10.18699/vjgb-26-10

**Published:** 2026-03

**Authors:** A.M. Mukhin, T.M. Khlebodarova, D.Yu. Oshchepkov

**Affiliations:** Kurchatov Genomic Center of ICG SB RAS, Novosibirsk, Russia Institute of Cytology and Genetics of the Siberian Branch of the Russian Academy of Sciences, Novosibirsk, Russia Novosibirsk State University, Novosibirsk, Russia; Kurchatov Genomic Center of ICG SB RAS, Novosibirsk, Russia Institute of Cytology and Genetics of the Siberian Branch of the Russian Academy of Sciences, Novosibirsk, Russia; Kurchatov Genomic Center of ICG SB RAS, Novosibirsk, Russia Institute of Cytology and Genetics of the Siberian Branch of the Russian Academy of Sciences, Novosibirsk, Russia

**Keywords:** phylogenetic footprinting, bacterial genome, transcription factor binding sites, motifs, bootstrap, Python, филогенетический футпринтинг, бактериальный геном, сайты связывания транскрипционных факторов, мотивы, бутстреп, Python

## Abstract

The rapid development of high-throughput sequencing technologies has led to an explosive accumulation of high-quality bacterial genome sequence data – their number is approaching three million, and this growth continues. This, in turn, provides additional impetus for the development of technologies for more efficient annotation using analytical methods designed to utilize such large-scale genomic data, as well as for achieving new levels of annotation quality. One such analytical approach is phylogenetic footprinting, which aims to identify motifs corresponding to transcription factor binding sites in the promoter regions of bacterial genomes by comparing corresponding sets of regulatory sequences of orthologous genes in related organisms. The continued accumulation of genomic data has served as the basis for further development of this approach. It has been found that an excessive number of sequences in a set analyzed using phylogenetic footprinting only reduces the accuracy of the method, whereas the inclusion of a sequence selection step in the analyzed set based on data on mutual evolutionary distances improves the method’s performance. In this paper, we propose and implement a further step in the development of the phylogenetic footprinting method. This step involves multiple runs of the selection step described above to generate distinct subsamples, subsequent pipeline runs for each subsample, and statistical analysis of the results obtained from multiple pipeline runs. The proposed approach, implemented in the MotifsOnFly method, improves the robustness of motif recognition results obtained from multiple pipeline runs. The effectiveness of the MotifsOnFly method is demonstrated using the analysis of the well-annotated promoter of the Escherichia coli OmpW gene

## Abstract (ru)

Активное развитие технологий высокопроизводительного секвенирования привело к взрывообразному накоплению высококачественных данных по последовательностям бактериальных геномов – их число приближается к трем миллионам, и дальнейший рост продолжается. Это, в свою очередь, дает дополнительный импульс развитию технологий для более эффективной их аннотации аналитическими методами с прицелом на применение таких больших геномных данных, а также получение нового качества аннотаций. Одним из таких аналитических подходов стал метод филогенетического футпринтинга, направленный на выявление мотивов, соответствующих сайтам связывания транскрипционных факторов в промоторных областях бактериальных геномов путем сравнения соответствующих выборок регуляторных последовательностей генов-ортологов для родственных организмов. Дальнейшее накопление геномных данных стало стимулом для развития подхода. Так, было обнаружено, что избыточное число последовательностей в выборке, анализируемой с использованием филогенетического футпринтинга, лишь ухудшает точность метода, тогда как включение этапа отбора последовательностей в анализируемую выборку с учетом данных о взаимных эволюционных расстояниях повышает качество работы метода. В настоящей статье нами предложен и реализован следующий шаг развития метода филогенетического футпринтинга, основанный на множественном запуске описанного выше этапа отбора для формирования различающихся подвыборок, последующего запуска конвейера для каждой из подвыборок и на статистическом анализе получаемых результатов множественных запусков конвейера. Предложенный подход, реализованный в методе MotifsOnFly, позволяет повысить устойчивость получаемых результатов распознавания мотивов, выявляемых в многократных запусках конвейера. Эффективность метода MotifsOnFly продемонстрирована на примере анализа хорошо аннотированного промотора гена OmpW Escherichia coli.

## Introduction

The rapid development and widespread application of highthroughput sequencing technologies in molecular genetics have stimulated advances not only in biotechnology – enabling large-scale assembly of bacterial genomes for analysis, modification, and subsequent utilization of bacterial strains to address biotechnological challenges – but also in bioinformatics methods for increasingly accurate genome annotation. Annotation of bacterial genomes with transcription factor binding sites (TFBSs) represents one of the most critical steps in biotechnology and microbiology, as the binding of transcription factors to their specific sites within gene promoters constitutes a fundamental mechanism regulating gene expression in bacteria (Browning, Busby, 2004).

The phylogenetic footprinting strategy, initially proposed in 1988 (Tagle et al., 1988; Katara et al., 2012) for identifying TFBSs, proved highly productive for de novo motif discovery, especially given the growing number of sequenced genomes available at the time. This strategy is based on the general principle that regulatory elements in promoters – such as TFBSs – are, in the vast majority of cases, evolutionarily more conserved and evolve at a slower rate at the DNA sequence level compared to surrounding non-functional sequences (Levy et al., 2001). The subsequent surge in the number of sequenced bacterial genomes further enhanced the effectiveness of phylogenetic footprinting (Blanchette, Tompa, 2002), spurring the development of numerous algorithms optimized for the volume of genomic data then available. These include MotifSuite, FootPrinter, AlignACE, BioProspector, CONSENSUS, MDscan, MEME, CUBIC, and BoBro (Hertz, Stormo, 1999; Liu X. et al., 2001, 2002; Blanchette, Tompa, 2003; Olman et al., 2003; Chen et al., 2008; Bailey et al., 2009; Li et al., 2011a; Claeys et al., 2012).

Later studies revealed that a limited number of properly selected reference promoters could be sufficient for identifying TFBSs within a gene (McCue et al., 2002). This is because closely related sequences with very short evolutionary distances provide little informative value for phylogenetic footprinting due to the insufficient accumulation of mutations in non-functional sequences flanking the TFBSs. Consequently, the continued accumulation of bacterial genomic data enabled refinement of the phylogenetic footprinting method by incorporating a step to pre-select promoter sequences for analysis based on their mutual evolutionary distances. This approach yields more informative sets of orthologous promoters for functional motif recognition. As demonstrated in (Liu B. et al., 2016), this refined methodology outperformed earlier popular motif-finding tools listed above in terms of TFBS prediction accuracy.

Building upon the approach proposed by B. Liu and colleagues (2016), we previously developed a computational pipeline for identifying TFBSs in bacterial genomes (Mukhin et al., 2024). This pipeline integrates a comprehensive suite of necessary databases and algorithms, enabling rapid annotation of selected bacterial genomes with transcription factor binding sites. However, during its application to identify TFBSs in the genome of Geobacillus icigianus (Peltek et al., 2024), we observed that the prediction outcome was sensitive to the order, composition, and selection method of the input promoter set. This dependency hindered definitive conclusions regarding the most probable TFBS location and the corresponding transcription factor likely interacting with it.

In the present study, we propose and implement a modification of our previously developed pipeline (Mukhin et al., 2024), which we call MotifsOnFly. This modification incorporates three key components: multiple runs of the pipeline’s sub-sampling stage, generating distinct promoter subsets from the full dataset while accounting for pairwise evolutionary distances to ensure diversity among subsets; execution of the full pipeline on each subset to identify overrepresented de novo motifs within each; and statistical analysis of the aggregated results. This bootstrap-like approach (with replacement)enables far more comprehensive utilization of the original data and significantly enhances the robustness and reliability of the results through statistical evaluation of motifs detected across multiple independent runs. Using the well-annotated promoter of the ompW gene in Escherichia coli as a case study, we demonstrate the advantages of the newly developed MotifsOnFly method, which stems directly from our proposed advancement of the phylogenetic footprinting strategy.

## Materials and methods

**Computational pipeline. **To identify potential transcription factor binding sites (TFBSs) by detecting de novo motifs in bacterial genomes, we employed a computational pipeline that leverages operon annotations for 3,850 bacterial genomes available in the DOOR2 database (Mao et al., 2014). The corresponding genomic sequences were retrieved from the NCBI database (Sayers et al., 2021).

The pipeline implements an integrative approach based on the phylogenetic footprinting method originally proposed by B. Liu and colleagues (2016). The implemented pipeline includes the following stages: for a given target gene, orthologous genes are identified across all 3,850 annotated genomes in the database by assessing protein sequence similarity using the GOST software module (Li et al., 2011b). For each identified orthologous gene, its promoter sequence is extracted based on the known operon structure of its host genome, thereby forming a complete set of orthologous promoter sequences for the target gene. Pairwise evolutionary distances between all promoters in this full set are estimated using a phylogenetic tree constructed with ClustalW2 (Larkin et al., 2007). Diverse promoter subsets are then generated by selecting sequences according to their mutual evolutionary distances, following the principles established in B. Liu et al. (2016). For each subset, de novo motif discovery is performed using a consensus (“voting”) approach that integrates results from multiple established motif-finding algorithms: AlignACE, BioProspector, CONSENSUS, MDscan, MEME, CUBIC, and BoBro (Hertz, Stormo, 1999; Liu X. et al., 2001, 2002; Olman et al., 2003; Chen et al., 2008; Bailey et al., 2009; Li et al., 2011a).

The discovered de novo motifs are compared against known TFBSs using the Tomtom algorithm (Gupta et al., 2007) and two reference databases: SwissRegulon (Pachkov et al., 2013), which contains curated TFBS data specifically for E. coli, and PRODORIC (Dudek, Jahn, 2022), a comprehensive resource for bacterial regulatory elements. Based on Tomtom’s statistical similarity scores, the best-matching known TFBS is identified, thereby predicting the most likely transcription factor (TF) interacting with the motif.

The use of both SwissRegulon and PRODORIC enables dual validation: for E. coli promoters, motifs can be matched against organism-specific TFBSs (SwissRegulon) as well as broader bacterial regulatory motifs (PRODORIC). Results from multiple pipeline runs undergo statistical analysis and visualization.All analytical scripts were implemented in Python versions 3.12, 3.6, and 2.7, with environment and dependency management handled via the Anaconda platform (https:// anaconda.com). Genomic data and structured metadata (genes, sequences, operons) were stored and managed using the PostgreSQL database system (https://www.postgresql.org/), deployed on the infrastructure of the Institute of Cytology and Genetics, Siberian Branch of the Russian Academy of Sciences (ICG SB RAS), by the multi-access center “Bioinformatics” ICG SB RAS.

**Visualization and analysis.** Known TFBS annotations for the E. coli genome were obtained from RegulonDB, the most comprehensive knowledgebase on transcription initiation regulation in Escherichia coli K-12 (Salgado et al., 2024). All analyses used the reference genome assembly NC_000913.3 from NCBI.

Genome annotation data (from NCBI), experimentally validated TFBSs (from RegulonDB), and projections of pipeline results onto the target promoter region were visualized using the modular genome browser JBrowse2 (Diesh et al., 2023), which supports a wide range of standard genomic file formats. The taxonomic relationships among the analyzed genomes – based on NCBI taxonomy (Sayers et al., 2021) – were visualized using the ETE3 toolkit (Huerta-Cepas et al., 2016). Statistical summaries and visual representations of multiple pipeline runs were generated using the Matplotlib library (Hunter, 2007).

## Results

**Modification of the phylogenetic footprinting approach implemented in the MotifsOnFly method**

**Fig. 1.**
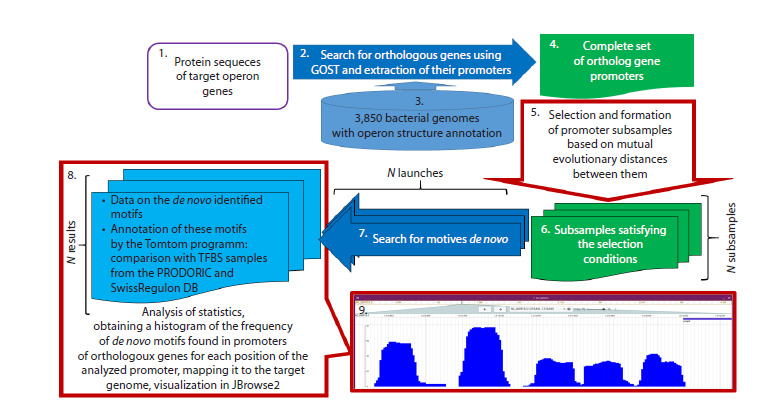
Schematic of the pipeline for de novo identification of functional motifs corresponding to transcription factor binding sites, based on the phylogenetic footprinting approach and implemented in the MotifsOnFly method. User-provided input data are indicated by a purple frame (1). Software modules (2 and 7) and external data sources (3) used in the pipeline are shown in blue. Intermediate data generated during pipeline execution are marked in green (4, 6). Steps specifically developed within the MotifsOnFly method are highlighted with a red border (5, 8, and 9). The stage involving selection and formation of promoter subsets based on pairwise evolutionary distances (5) enables multiple runs of the pipeline to perform de novo motif discovery (7) on these distinct subsets. The resulting data – including identified de novo motifs (8) and statistics on their positional distribution across aligned orthologous promoters – allow generation of a histogram showing the frequency of detected de novo motifs, which is visualized in the JBrowse2 genome browser (9). Furthermore, each de novo motif obtained from individual runs is compared using the Tomtom tool against known TFBS databases – SwissRegulon and PRODORIC (8) – and subsequent statistical analysis of these comparisons enables prediction of the most likely transcription factor regulators of the target promoter.

The modification of the approach implemented in this study is based on multiple runs of the pipeline stage responsible for generating diverse subsets of promoter sequences, while preserving the selection principles previously demonstrated to be effective (Liu B. et al., 2016), and accumulation and statistical analysis of the results obtained from these repeated runs on the generated subsets. The conceptual workflow of the analysis implemented in the developed MotifsOnFly pipeline is shown in Figure 1. In accordance with the pipeline steps described in detail in the “Materials and methods” section, the protein sequence of the target gene is provided as input to the pipeline (1). This triggers the identification of orthologous genes using the GOST software module (2) across the set of annotated genomes available in the database (3). Subsequently, utilizing the same genomic sequences along with their operon structure annotations (3), promoter sequences of all identified orthologous genes are extracted (4).

The core of the developed modification is the stage of generating multiple promoter subsets (5), while preserving the selection criteria based on pairwise evolutionary distances among promoters. The effectiveness of using such selection criteria in phylogenetic footprinting was previously demonstrated (Liu B. et al., 2016). The full set of promoters (4), ordered by their evolutionary distances from the target promoter (calculated using ClustalW2), was divided into three subgroups with distances ranging from 0.05 to 0.31, from 0.31 to 0.55, and from 0.55 to 0.73, respectively. Based on the sizes of these subgroups, the maximum number M of subsets was determined such that all promoters from the full set could be distributed into subsets of 12 promoters each, maintaining a fixed proportion of 3:3:6 (i. e. 3 promoters from the first subgroup, 3 from the second, and 6 from the third). In the final step, N = 2M subsets were generated through random sampling according to the 3:3:6 proportion, along with additional selection rules that consider pairwise distances among sequences within each subset, thereby preventing the inclusion of overly similar promoters. This bootstrap-like approach (with replacement) ensures maximal – but not exhaustive – inclusion of promoters from the original set into the analysis, as adherence to selection principles that exclude low-information promoters remains a priority for method efficacy (McCue et al., 2002).

The resulting subsets, each supplemented with the target promoter (5), are used for repeated runs of the de novo motif discovery stage (7), as detailed in the “Materials and methods” section. Across these multiple runs, statistical data (8) are accumulated, recording which de novo motifs were identified at which positions in the target promoter and simultaneously detected in orthologous promoters. These aggregated data yield a histogram showing the frequency of detected de novo motifs at each position of the target promoter across orthologous sequences, enabling assessment of motif reliability at each site (Tompa et al., 2005). The histogram is saved in BigWig format for subsequent visualization in the JBrowse2 genome browser (9).

**Analysis of the ompW gene promoter in Escherichia coli K-12 using the developed MotifsOnFly method**

The best-studied and most comprehensively annotated bacterial genome to date remains that of E. coli K-12 (Salgado et al., 2024). To demonstrate the capabilities of our newly developed MotifsOnFly method – which extends the approach originally implemented by B. Liu and colleagues (2016) – we selected the ompW gene of E. coli. The expression of this gene is regulated by six transcription factors acting through five known binding sites. The ompW gene encodes an outer membrane protein and exhibits a broad range of physiological functions, including bacterial resistance to various antibiotics and herbicides, tolerance to osmotic stress, and support of bacterial growth under harsh environmental conditions such as hypoxia and elevated temperature (Zhang et al., 2020). A distinctive feature of the promoter of this operon – which contains only the ompW gene – is the presence of both isolated transcription factor binding sites (TFBSs) and clusters of overlapping TFBSs for different transcription factors. As input for the pipeline, we used the protein and promoter sequences of the gene, as described in the “Materials and methods” section.

**Fig. 2.**
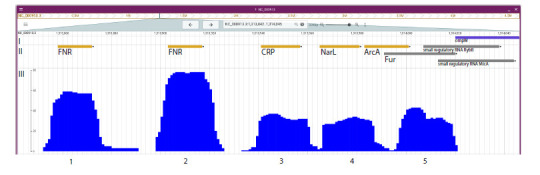
Screenshot of the JBrowse2 genome browser. I – genome annotation in the ompW promoter region according to NCBI data; II – experimentally validated transcription factor binding site (TFBS) locations from the RegulonDB database; III – pipeline output: a histogram showing the frequency of de novo motifs detected at each position of the analyzed promoter across orthologous sequences. The histogram displays distinct peaks, numbered in accordance with their reference in the main text.

Figure 2 shows the pipeline results visualized using JBrowse2, including: (I) genome annotation in the ompW gene region according to NCBI data; (II) experimentally validated TFBS locations from the RegulonDB database; and (III) a histogram displaying the frequency of de novo motifs detected at each position of the analyzed promoter across orthologous sequences. The histogram reveals distinct peaks (Fig. 2), indicating positions in the ompW promoter where de novo motifs were consistently identified across a substantial proportion of pipeline runs. Specifically: peaks 1 and 2 coincide with experimentally confirmed binding sites for the transcription factor FNR; peak 3 coincides with the known binding site for the transcription factor CRP; peak 4 overlaps with the experimentally verified binding site for the transcription factor NarL; peak 5 overlaps with binding sites for both ArcA and Fur, illustrating the complexity of deciphering regulatory signals near the transcription start site and warranting further dedicated analysis. Additionally, a noticeable “tail” to the right of peak 1 indicates that de novo motifs were also detected in this region during some pipeline runs. This observation confirms the instability of results obtained from single-run analyses and underscores the value of the MotifsOnFly method’s multi-run, statistically robust framework.

**Fig. 3.**
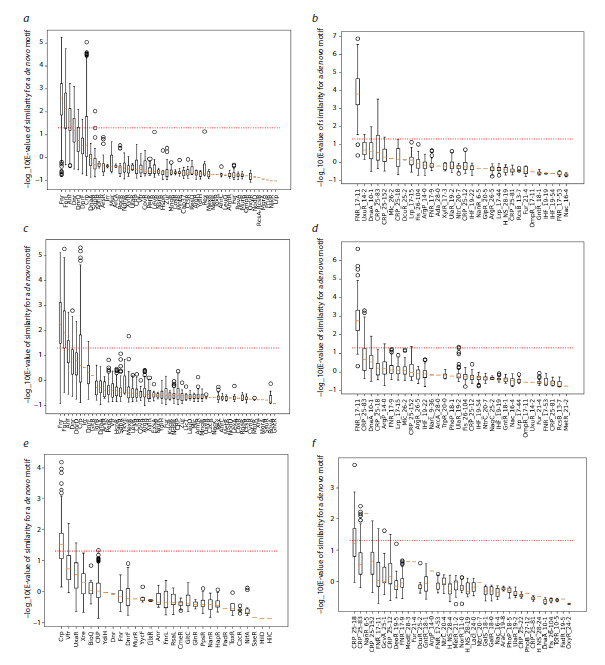
Box-and-whisker plots showing the statistics of comparisons between the identified motifs for peak 1 (a, b), peak 2 (c, d), and peak 3 (e, f) against known TFBS databases PRODORIC (a, c, e) and SwissRegulon (b, d, f). The red dashed line indicates the 5 % significance threshold. Statistical analysis results from multiple pipeline runs were visualized using the Matplotlib software package.

Comparison of the identified de novo motifs with known transcription factor binding sites (TFBSs) from the SwissRegulon and PRODORIC databases using the Tomtom tool allows prediction of the most likely transcription factor regulators of the analyzed promoter. For each such comparison, an E-value is calculated to assess the statistical significance of the similarity between the discovered motif and each known TFBS. We computed E-value statistics for all candidate transcription factors corresponding to all peaks (Fig. 3). As evident from the charts, this statistical analysis enables unambiguous identification of FNR as the most probable transcription factor binding to peaks 1 (Fig. 3a, b) and 2 (Fig. 3c, d), and CRP for peak 3 (Fig. 3d, e), regardless of which database is used.

**Fig. 4.**
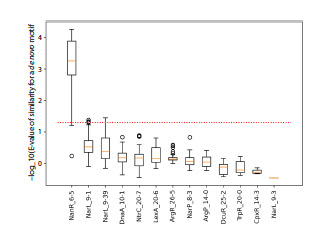
Box-and-whisker plot showing the statistics of comparisons between the identified motifs for peak 4 and known transcription factor binding sites (TBFSs) from the SwissRegulon database The red dashed line indicates the 5 % significance threshold. Results of the statistical analysis from multiple pipeline runs were visualized using the Matplotlib software package.

For peak 4, significant matches were found only with sites from the SwissRegulon database (Fig. 4), specifically for the transcription factors NanR and NarL. For peak 5, no significant matches with known TFBSs were obtained from either database, suggesting the need for further in-depth analysis.

It should be noted that for peak 1, in the case of a single pipeline run, FNR would be identified as the top candidate in only 72 % of cases when using the SwissRegulon database and in just 43 % of cases with PRODORIC. Similarly, for peak 2, correct identification of FNR occurs in 90 and 65 % of single runs (SwissRegulon and PRODORIC, respectively), while for peak 3, CRP is correctly prioritized in only 49 and 54 % of cases, respectively. Our proposed modified approach overcomes this instability inherent in single-run phylogenetic footprinting analyses and enables unambiguous prioritization of results that fully align with experimental data.

**Fig. 5.**
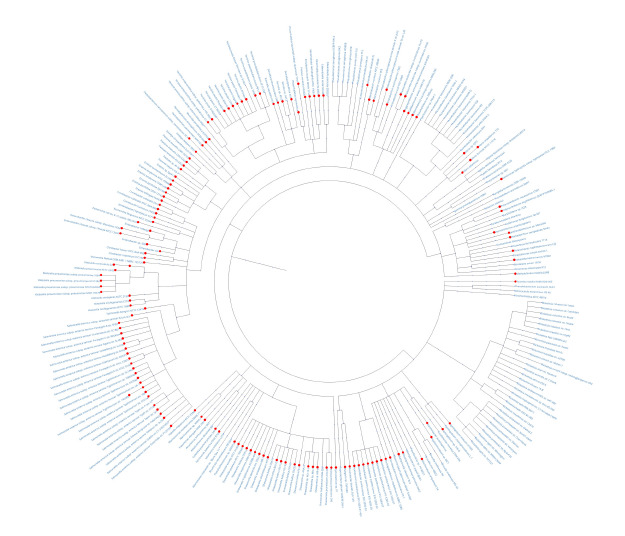
Visualization of the presence of similar motifs (indicated by red dots) in the promoters of orthologous genes from various bacterial species at positions corresponding to peak 1 in the alignment with the target promoter. The tree was constructed using taxonomic information for the analyzed genomes as provided by NCBI. Tree visualization was performed with the ETE3 package (Huerta-Cepas et al., 2016).

Moreover, the method allows tracking the presence of de novo motifs corresponding to each peak across all orthologous promoters and visualizing this information on the taxonomic tree of the genomes analyzed. Specifically, if a similar motif is detected in the promoter of an orthologous gene from a given bacterial species at the position aligned with a peak in the target promoter, this is indicated by a red dot on the corresponding node of the tree. As an example, Figure 5 illustrates the occurrence of such similar motifs in orthologous promoters at positions aligned with peak 1 of the target promoter.

**Fig. 6.**
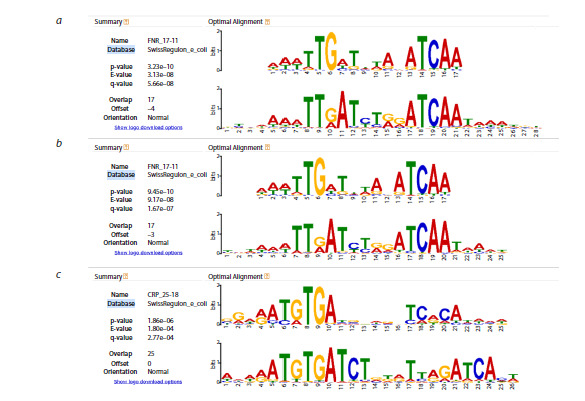
Results of the comparison between the consolidated alignments of peak regions 1–3 and known transcription factor binding sites. For each peak, a screenshot of the best match from the SwissRegulon database, as reported by the Tomtom tool, is shown. The alignments exhibit statistically significant similarity to FNR binding sites for peak 1 (a, E-value = 3.13 × 10–8), FNR binding sites for peak 2 (b, E-value = 9.17 × 10–8), and CRP binding site for peak 3 (c, E-value = 1.80 × 10–4). These results are in complete agreement with experimental data.

The information enabling tracking of similar motifs across all orthologous promoters (Fig. 5) within a single peak also allows the construction of a consolidated alignment that includes only those orthologous promoters in which a similar motif was detected simultaneously with the target promoter in at least one pipeline run. Thus, this approach yields a consolidated alignment of all conserved DNA segments corresponding to a given peak, ensuring maximal representation of biologically relevant sequences in the resulting alignment. The boundaries of these informative aligned regions are naturally defined by the width of each peak, which served as the criterion for selecting the length of the consolidated alignment. This strategy also incorporates flanking sequences adjacent to the core binding sites, thereby improving the quality and sensitivity of comparisons with known TFBS databases. These consolidated alignments for each peak were subsequently analyzed using the Tomtom tool. Comparison of the consolidated alignments for peaks 1–3 against known TFBSs yielded the highest confidence matches when using the SwissRegulon database (Fig. 6). In full agreement with experimental data, the top-scoring candidates were again FNR for peak 1 (Fig. 6a) and peak 2 (Fig. 6b), and CRP for peak 3 (Fig. 6c), with E-values of 3.13 × 10–8, 9.17 × 10–8, and 1.80 × 10–4, respectively. These results are consistent with the earlier statistical analysis of individual motif comparisons (Fig. 3) but demonstrate substantially higher confidence in motif similarity due to the increased representativeness and signalto- noise ratio achieved through the consolidated alignments.

**Fig. 7.**
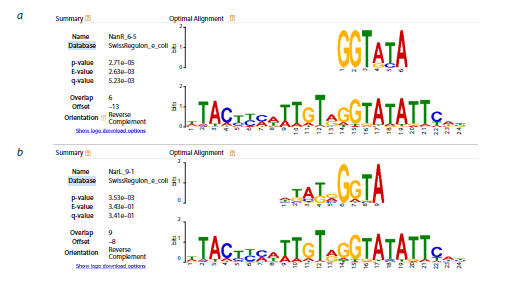
Comparison of the consolidated alignment for the peak 4 region Screenshots from the Tomtom output against the SwissRegulon database are shown. The alignment exhibits significant similarity to the NanR binding site (a) and non-significant similarity to the NarL binding site (b).

Tomtom analysis of the consolidated alignment for peak 4 yielded significant results only when using the SwissRegulon database: a statistically significant similarity was found with the NanR binding site (Fig. 7a), while similarity to the NarL binding site was not statistically significant (Fig. 7b). No other transcription factors with binding sites resembling peak 4 were identified.

**Fig. 8.**
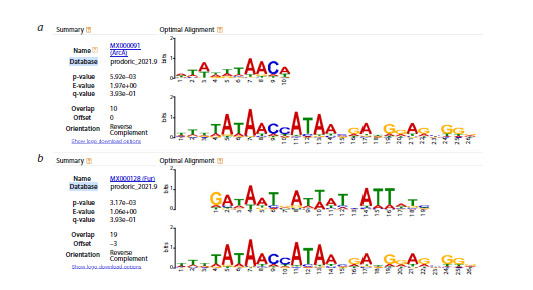
Comparison of the consolidated alignment for the peak 5 region. Screenshot from the Tomtom output against the PRODORIC database is shown. Weak (non-significant) similarities are observed between parts of the consolidated alignment for peak 5 and the binding sites of ArcA (a) and Fur (b). Notably, the predicted binding sites for these two transcription factors exhibit substantial overlap in this region.

The consolidated alignment for the peak 5 region, when analyzed with Tomtom, yielded results only upon comparison with the PRODORIC database. However, none of the transcription factor binding sites showed statistically significant similarity to this alignment. Nevertheless, weak (non-significant) similarities were observed with the binding sites of ArcA and Fur (Fig. 8a, b), the known binding sites of which substantially overlap in this genomic region.

## Discussion

We have developed a computational pipeline for identifying functional motifs corresponding to transcription factor binding sites (TFBSs), based on a modification of the phylogenetic footprinting method originally proposed by B. Liu and colleagues (2016). The core idea of our modification lies in repeated execution of an effective promoter selection procedure that accounts for pairwise evolutionary distances, thereby generating diverse promoter subsets. The pipeline is then run independently on each subset, and the results from all runs undergo statistical aggregation (Fig. 1). The approach implemented in our MotifsOnFly method employs a bootstrap-with-replacement scheme, enabling maximal utilization of currently available genomic data while simultaneously leveraging statistical robustness across varying promoter subsets

The effectiveness of the pipeline was demonstrated through the analysis of the ompW gene promoter – one of the most comprehensively annotated promoters in Escherichia coli K-12. This gene encodes an outer membrane protein and is known to be regulated by six transcription factors, with five experimentally validated binding sites (Salgado et al., 2024). A detailed regulatory map of this promoter was previously described (Xiao et al., 2016). Our pipeline generated a histogram showing the frequency of de novo motifs detected at each position of the target promoter across orthologous sequences (Fig. 2). This histogram revealed distinct peaks that correspond precisely to experimentally confirmed TFBSs, thereby providing reliable identification of conserved, motif-enriched promoter regions. Thus, our approach vividly illustrates the essence of phylogenetic footprinting – highlighting the most evolutionarily conserved segments within alignments of orthologous promoters.

Statistical analysis of de novo motifs identified across multiple pipeline runs, followed by comparison against known TFBSs from SwissRegulon and PRODORIC, enabled confident assignment of the most likely regulatory factors. Predictions made by MotifsOnFly fully aligned with experimental data for peaks 1, 2, and 3: FNR (Constantinidou et al., 2006; Myers et al., 2013; Xiao et al., 2016) was correctly identified as the regulator for peaks 1 and 2, and CRP (Gaston et al., 1990; Ushida, Aiba, 1990; Xiao et al., 2016) for peak 3 (Fig. 3). Importantly, however, single-run analyses often failed to yield the correct top candidate – only 72 (SwissRegulon) or 43 % (PRODORIC) of single runs correctly prioritized FNR for peak 1, for example. This underscores the instability of single-run phylogenetic footprinting, which our multi-run framework successfully overcomes. Moreover, by constructing consolidated alignments of all conserved motif instances corresponding to each peak and comparing them to reference databases (Fig. 6), we achieved extremely high-confidence matches, with E-values as low as 3.13 × 10–8. Such high-quality alignments not only confirm known regulatory interactions but also open the possibility of generating reliable motif models for TFBSs that are underrepresented in current databases. The pipeline also includes functionality to track the presence of de novo motifs across all analyzed orthologous promoters and visualize this information on a taxonomic tree (Fig. 5) – a feature valuable for studying the evolutionary conservation and distribution of regulatory signals.

An intriguing result emerged for peak 4 (Fig. 4 and 7). Although experimental data indicate a NarL binding site in this region (Tyson et al., 1993, 1994; Xiao et al., 2016), both the statistical analysis of multiple runs and the consolidated alignment point to NanR as the top-scoring TF, with NarL ranked second. The consensus NarL site “TACYYMT” does show similarity to the peak 4 alignment, yet our highest-confidence match corresponds to the NanR motif GGTATA (Fig. 7). However, this finding warrants caution: NanR functions as a dimer, and high-affinity DNA binding requires three adjacent GGTATA repeats for cooperative binding of three NanR dimers (Kalivoda et al., 2013; Horne et al., 2021). The presence of only a single GGTATA instance at peak 4 casts doubt on NanR’s biological relevance in regulating ompW. Furthermore, NanR’s regulatory role in E. coli is currently considered to be minor, limited to four operons involved in sialic acid catabolism (Kalivoda et al., 2013; Shimada et al., 2018). Nevertheless, the possibility of ompW (or some orthologs) being regulated via this NanR-like site merits further experimental investigation.Regarding peak 5, it fully overlaps with the σ70 RNA polymerase binding region, encompassing the transcription start site and canonical −35/−10 promoter elements. This area is densely packed with regulatory signals: RegulonDB (Salgado et al., 2024) annotates overlapping binding sites for ArcA (Park et al., 2013; Xiao et al., 2016) and Fur (Zhang et al., 2020). The structural plasticity of ArcA binding sites allows them to coexist with other TF motifs in compact sequence space (Park et al., 2013). Meanwhile, the Fur site in this region is considered weak (Zhang et al., 2020), possibly due to competitive interactions with SoxS, which can bind the −35 element under oxidative stress conditions induced by iron-bound Fur (Graham et al., 2012; Taliaferro et al., 2012). Thus, the promoter region of the ompW gene in the vicinity of peak 5 possesses substantial regulatory potential. Notably, our analysis did not identify any strong similarity between the peak 5 consolidated alignment and known TFBSs in either database. This may reflect the absence of strong, obstructive TFBSs that would interfere with σ70 RNA polymerase binding – consistent with the need for basal transcription. At the same time, the alignment shows comparable, low-significance similarity to numerous TFBSs in PRODORIC (Fig. 8), suggesting the presence of multiple weak, overlapping sites, including those for ArcA and Fur. This interpretation aligns well with existing literature and highlights the complex, layered regulatory logic operating near the core promoter.

## Conclusion

The modification of the phylogenetic footprinting approach proposed in our work and implemented in the MotifsOnFly method represents a natural evolution of the strategy developed by B. Liu and colleagues (2016), designed to address the continuously growing volume of large-scale genomic data. MotifsOnFly enables multiple pipeline runs on diverse subsets of orthologous promoter sequences, yielding refined localization of transcription factor binding sites (TFBSs) and facilitating robust statistical analysis. The forms of such statistical analysis are not limited to those described in this article and can be further extended according to the specific goals and requirements of individual research projects. As demonstrated here, the MotifsOnFly method produces reliable and stable TFBS predictions, making it a valuable tool for a broad community of researchers engaged in the annotation and analysis of regulatory sequences in bacterial genomes.

## Conflict of interest

The authors declare no conflict of interest.
